# Analyzing temporal imaging patterns in acute ischemic stroke via DICOM-timestamps

**DOI:** 10.1038/s41598-025-85315-5

**Published:** 2025-01-07

**Authors:** Alexander Rau, Marco Reisert, Benedikt Frank, Cornelius Deuschl, Maximilian F Russe, Samer Elsheikh, Martin Köhrmann, Horst Urbach, Elias Kellner

**Affiliations:** 1 Department of Neuroradiology, Medical Center – University of Freiburg, Faculty of Medicine, University of Freiburg, 79106 Freiburg, Germany; 2 Medical Physics, Department of Diagnostic and Interventional Radiology, Medical Center – University of Freiburg, Faculty of Medicine, University of Freiburg, 79106 Freiburg, Germany; 3 Department of Neurology and Center for Translational Neuro- and Behavioral Sciences (C-TNBS), University Hospital Essen, 45147 Essen, Germany; 4 Faculty of Medicine, Institute of Diagnostic and Interventional Radiology and Neuroradiology, University Hospital Essen, Hufelandstrasse 55, 45147 Essen, Germany; 5 Department of Diagnostic and Interventional Radiology, Medical Center – University of Freiburg, Faculty of Medicine, University of Freiburg, 79106 Freiburg, Germany

**Keywords:** DICOM-timestamps, Acute ischemic stroke, Process management, Medical imaging, Epidemiology, Stroke

## Abstract

Acute stroke management is time-sensitive, making time data crucial for both research and quality management. However, these time data are often not reliably captured in routine clinical practice. In this proof-of-concept study we analysed image-based time data automatically captured in the DICOM format. We enrolled data from two separate stroke centers (*n* = 3136 and *n* = 2089). Data from the first center was additionally separated into groups with large-vessel-occlusion (LVO, *n* = 1.092), medium-vessel-occlusions (MVO, *n* = 416), and no occlusion (NVO, *n* = 1630). The DICOM-tag *StudyTime* was used to analyze the distribution of scan times throughout the day. Additionally, manually documented onset- and admission were extracted from the patients’ records in a subset of cases (*n* = 347). Timestamps were compared across centers and occlusion groups, and a probabilistic model was developed to illustrate and compare stroke occurrence patterns throughout the day. The temporal distribution of the scan times at both centers was exceptionally consistent with a peak around noon and a nighttime low. The groups with vessel occlusions showed an earlier peak compared to those without (*p* < 0.04). The median interval between admission and scan time was 23 min, while the median onset-to-imaging time was 1 h:54 min. This proof-of-concept study indicates that DICOM-timestamps can reveal insights into the temporal patterns of stroke imaging and may be a promising tool for quality control and stroke research in general since they are always automatically captured by imaging devices as opposed to manual data collection in routine clinical practice.

## Introduction

The management of patients with an acute stroke is time crucial, as persistent malperfusion leads to an irreversible loss of neurons due to their low tolerance to ischemia, as “time is brain”^1^. Ischemia can be successfully treated by both intravenous thrombolysis (IVT) and endovascular thrombectomy (EVT), but the therapeutic success is highly time-dependent^2,3^. Correspondingly, diagnostics and therapy need to be provided as fast as possible in an acute stroke.

To improve the treatment and workflows and understand pitfalls, statistical data on the timeline are highly relevant for both quality management and research purposes^4^ and international guidelines recognize that “establishing and monitoring target time goals […] can be beneficial to monitor and enhance system performance”^5^. However, these statistical time data are often not reliably and standardized documented in clinical routine^6^.

In this respect, it is desirable to use a time stamp that is as standardized as possible and captured in an automatic manner. One approach in this context might be based on the exploitation of imaging-related time data as the diagnostic workup of acute stroke symptomology includes neuroimaging. Imaging data is consistently stored in the DICOM (*Digital Imaging and Communications in Medicine*) format, which always contains time-related data such as *StudyTime* in the file headers^7,8^. Due to the strict standardization, these data are always available and can be read automatically and retrospectively from any PACS database.

In a comprehensive examination of crucial stages in the stroke care workflow, prior studies have theoretically proposed using DICOM metadata as a tool for analysis and optimization^4^. In this proof-of-concept study, we evaluated the feasibility of using imaging-derived timestamps for research and quality control purposes in stroke care. We retrospectively analyzed automatically captured DICOM-based time data and manually recorded onset- and admission times from two separate stroke centers in Germany, creating histograms to chart the distribution of scan times throughout the day. We investigated: (1) Whether the time intervals between onset, admission, and scan times followed a consistent sequence (2) Whether there was consistency in the distribution of scan times among the two separate stroke centers. (3) Whether scan times differed between patients with vessel occlusions and those without. (4) Whether the overall distribution of scan times could be explained by a probabilistic model based on sleep and wake-up times and transfer to the hospital.

## Methods

### Patients

For this retrospective study, we enrolled *n* = 5,225 patients from two separate tertiary referral stroke centers that received multimodal CT or MR including perfusion-imaging due to an acute ischemic stroke between 02/2011 and 02/2022 (*n* = 3,136 in center 1 and *n* = 2,089 in center 2). The study was approved by the Institutional Review Board (Ethics Committee – University of Freiburg; EK 20/1047) and carried out in accordance with the Declaration of Helsinki and its later amendments. Due to the retrospective nature of this study, the need for written informed consent was waived (Ethics Committee – University of Freiburg; EK 20/1047).

### Assessment of vessel occlusion

To determine whether patients with large vessel occlusions (LVO) present a different temporal distribution of imaging compared with medium vessel occlusions (MVO) or no vessel occlusions (NVO), we assessed these subgroups separately. For this purpose, data from the first center i.e. documented clinical reports along with the angiography and perfusion analysis - the latter calculated with the VEOcore software^9^ - were analyzed. An LVO was defined as occlusion of the internal carotid artery (ICA), first and second segments of the middle cerebral artery (MCA, M1, M2), the anterior cerebral artery, vertebral artery, basilar artery, or the proximal posterior cerebral artery^10^. An MVO was defined when the perfusion maps showed a hypoperfused territory but large vessels were open. When CTA and perfusion maps showed no abnormality the scan was assessed as NVO.

### Extraction of DICOM-Tags

The DICOM headers contain multiple time-related attributes such as *StudyTime*,* SeriesTime*,* ContentTime*, and others. For the present analysis, we chose *StudyTime* which reflects the time point when the patient is registered on the scanner´s console and thus provides the earliest possible timestamp in the imaging workflow. All tags were extracted from the PACS systems using C-FIND command.

### Extraction of onset- and admission times

For patients at center 1, patient records were reviewed to extract manually documented onset and admission times, when available.

### Data analysis

Median values of the onset-to-scan and admission-to-scan intervals were calculated. The DICOM timestamps data were evaluated from two perspectives: An empirical one, and a model-based perspective. For empirical comparison, histograms were plotted and compared visually. A Kolmogorov-Smirnov test (KS-test) was performed to test the null hypothesis of equal distribution for (a) center 1 versus center 2 and (b) LVO, MVO, and NVO. The significance threshold was set to *p* < 0.05. For a model-based analysis, we employed the following probabilistic model for illustrative and comparative purposes.

### Probabilistic model

**Fig. 1:**
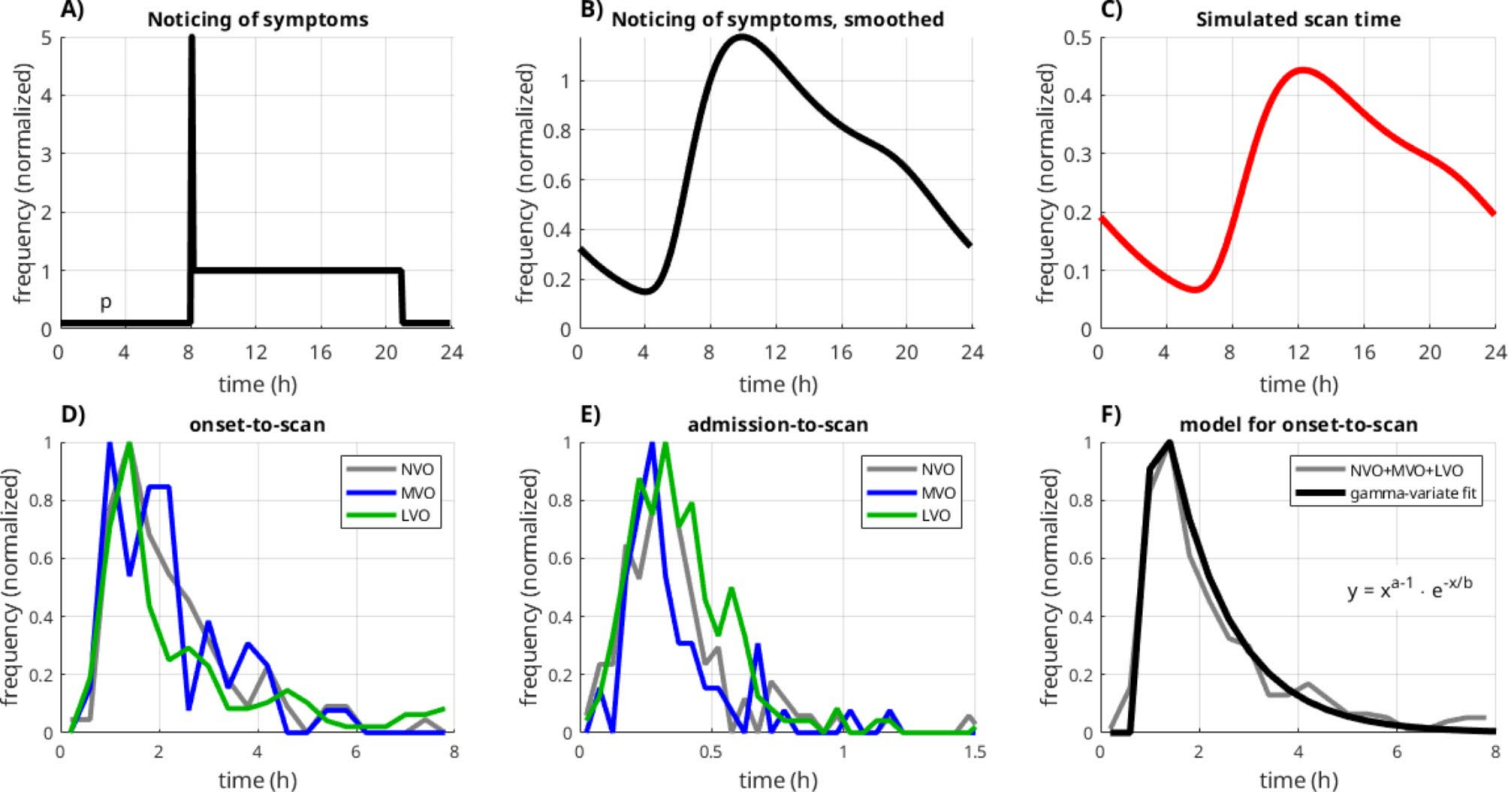
Construction of the probabilistic model is depicted in (**A**–**C**). The model for the time when symptoms are noticed has a chair-like shape (**A**), where the chairback corresponds to the wake-up-strokes. In order to account for some variance in this idealized model, the chair function is smoothed with a gamma-variate-function (**B**). Subsequently, the delay from noticing symptoms until imaging in the hospital is incorporated with another gamma-variate function with parameters derived from a fit to the actually documented onset-to-scan intervals (**F**). This chain of convolutions finally yields the model for the scan time distributions (**C**). Panels (**D**–**F**) shows the sub-analysis of onset, admission and scan times in a subset of patients. The respective distributions demonstrate a similar pattern for the subgroups with no significant difference between NVO, MVO and LVO. A reasonable gamma-variate curve fit to the onset-to-scan intervals could be obtained (**F**).

The construction of the probabilistic model for the scan times is illustrated in (Fig. 1). For the stroke occurrence itself (i.e. the vessel occlusion), we assumed a uniform probability throughout the day, i.e. we neglected a circadian variation. For the sleeping pattern, we assumed that, on average, individuals go to sleep around 21:00 and wake up around 8:00. Further, we assumed that some individuals may be awake during the night with a certain probability p. Based on this, two situations can be distinguished for the onset time (i.e. the timepoint when symptoms are noticed and an ambulance is called): The person can either be awake when the stroke occurs, or only notice the symptoms after waking up (“wake-up-strokes”), with unknown onset times. The resulting function looks similar to a “chair”, in which the chairback accounts for the wake-up strokes (Fig. 1A). To introduce variability in this idealized and simplified model, the distribution was convolved with a gamma-variate function^11^, denoted g1 in the following, which results in a smoothed distribution (Fig. 1B). Finally, the time interval from onset to imaging - encompassing emergency medical service and transport to the hospital was incorporated using another gamma-variate function g2. With this final model (Fig. 1C), nonlinear fitting was performed. For a reasonable fit stability, parameters for g2 were set to fixed values derived from the onset-to-scan distribution actually measured in a subset of patients (Fig. 1E) and all other parameters were left free. We calculated the proportion of wake-up strokes from the fitted probability p described above and characterized the gamma variate functions in terms of peak position and full-width-half-maximum.

To assess the relative robustness of the fitted parameters, we calculated the standard deviation of repeated fitting (*n* = 50) with the addition of Gaussian noise (sigma = 2.0 h) to the timestamps.

### Analysis of StudyDate

As a secondary analysis, we examined monthly and weekly distributions in imaging scans by analyzing the day-of-week and month-of-year distributions using the DICOM tag *StudyDate*. Month-of-year distributions were calculated based on full years 2012–2021 and normalized for different numbers of days within a month (e.g., February only has 28 days).

### Data processing tools

For data management, image processing and image reading, a local instance of the imaging platform NORA (www.nora-imaging.org*)* was used. Model fitting and statistical analysis was performed using MATLAB (the MathWorks, Natick, USA).

## Results

### Comparison of onset- admission- and imaging times

A full set of onset-, admission- and scan times could be extracted in a subset of 347 cases from center 1. Median values of calculated onset-to-scan intervals and admission-to-scan intervals were 1 h:54 min, and 23 min, respectively. The respective distributions for the time intervals are shown in Fig. 1D,E and demonstrate a similar pattern for the LVO, MVO, and NVO subgroups (*n* = 183, *n* = 61, and *n* = 103, respectively). The KS-test did not reveal any significant differences between the subgroups. A reasonable gamma-variate to the onset-to-scan intervals could be obtained (Fig. 1F), which was used in the probabilistic model.

### Comparison of imaging times between the two centers

**Fig. 2:**
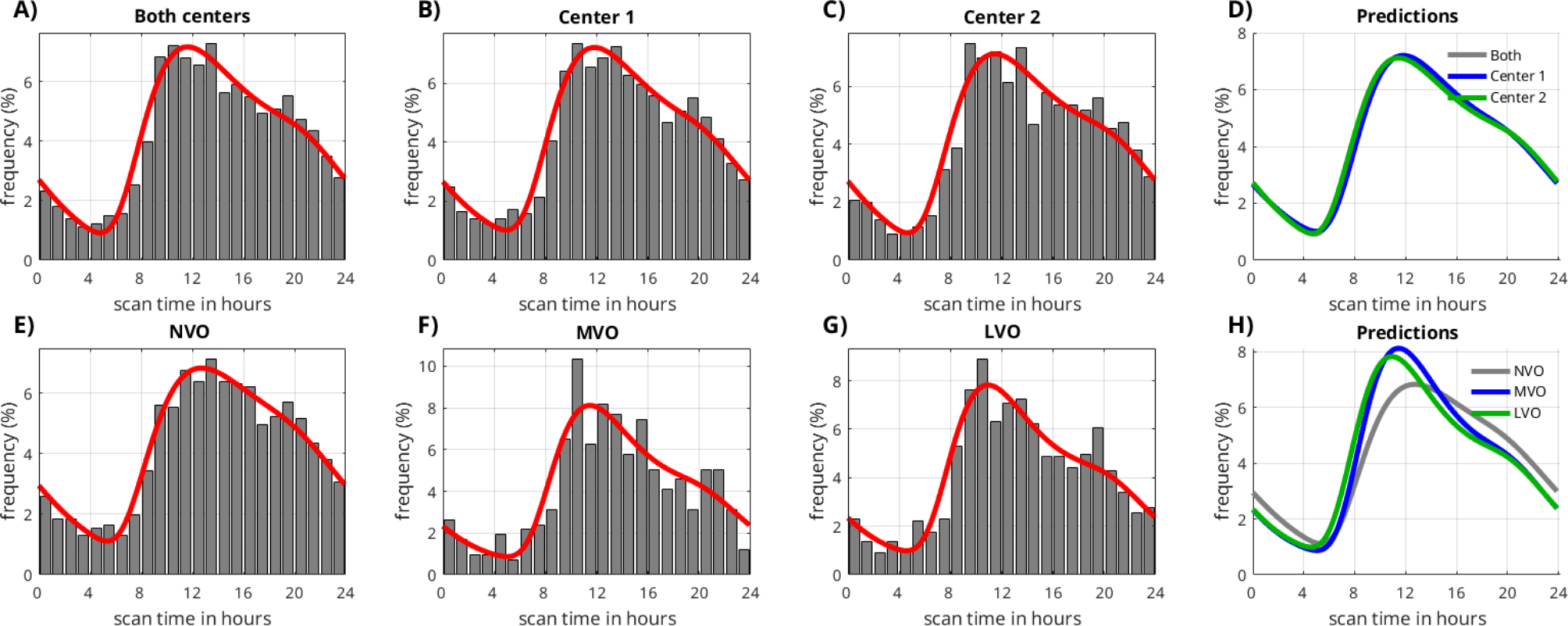
Hour-wise distribution of scan times (normalized to the area-under-curve). (**A**–**D**) Distributions from two separate stroke centers are very consistent with a peak around noon. The empirical histograms align well with the model’s predictions. (**E**–**H**) The sub-analysis of large and medium vessel occlusions revealed an earlier and sharper peak for the large vessel occlusion (LVO) and medium vessel occlusion (MVO) as compared to the group with no vessel occlusion (NVO).

Data from 3,136 cases (center 1) and 2,089 cases (center 2) were assessed both pooled and individually for the respective center. The empirically derived temporal distributions along with the fitted model functions are shown in (Fig. 2). All histograms present a similar shape, with a peak around noon (11 am) and a nighttime reduction with a minimum around 5:00 am. The distributions of center 1 and center 2 (Fig. 2B and C) are almost identical and the KS-test indicated no significant difference (*p* = 0.069; Fig. 2D).

### Comparison between subgroups with and without vessel occlusion

From the 3,136 cases of center 1, patients had an LVO in 1,091 cases, an MVO in 416 cases, whereas NVO was documented in 1,629 cases. The histograms of all groups are similar in shape, but the LVO and MVO groups show an earlier and sharper peak (Fig. 2E–H). While the LVO and MVO groups showed a very similar temporal distribution with no significant difference (*p* = 0.81), KS-test indicates a significant difference for both MVO and LVO vs. NVO (*p* = 0.04 and *p* = 0.01, respectively).

### Probabilistic model

A good fit of the probabilistic model and empiric real-world data was achieved in all settings (i.e. center 1, center 2, NVO, MVO, and LVO). Reasonable bed- and rise times around 21:00 and 8:30 were obtained. The calculated proportion of wake-up-strokes ranged between 40 and 50%. Details are provided in (Table 1).

**Table 1:** Results from curve fitting of the probabilistic model to the empiric real-world data, as illustrated in (Fig. 2). Parameters a and b are mathematical parameters of the gamma-variate function (formula see Fig. 1C). Time of maximum (T) and full-width-half-maximum (FWHM) were derived from the fitted functions as more descriptive parameters. Standard deviations were obtained by repeated fitting (*n* = 50) with gaussian noise (sigma = 2.0 h).

	Gamma-variate g1 parameters				*p*	Bed-time	Rise-time	Proportion of wake-up strokes
	A	B	T	FWHM				
Center 1	3.2 ± 0.1	2.0 ± 0.1	4.5 ± 0.1	7.0 ± 0.4	0.07 ± 0.03	21.2 ± 0.0	8.5 ± 0.2	44% ± 2%
Center 2	3.3 ± 0.1	2.0 ± 0.1	4.5 ± 0.1	6.9 ± 0.5	0.00 ± 0.02	21.7 ± 0.0	8.1 ± 0.1	43% ± 3%
NVO	2.8 ± 0.2	2.6 ± 0.1	4.6 ± 0.2	8.3 ± 0.3	0.03 ± 0.02	21.2 ± 0.1	9.0 ± 0.3	48% ± 4%
MVO	4.0 ± 0.5	1.4 ± 0.4	4.2 ± 0.1	5.8 ± 1.0	0.13 ± 0.10	20.8 ± 0.2	8.5 ± 0.2	43% ± 4%
LVO	4.0 ± 0.2	1.4 ± 0.3	4.2 ± 0.1	5.8 ± 0.4	0.14 ± 0.06	20.8 ± 0.1	7.9 ± 0.3	40% ± 3%

### Analysis of StudyDate

**Fig. 3:**
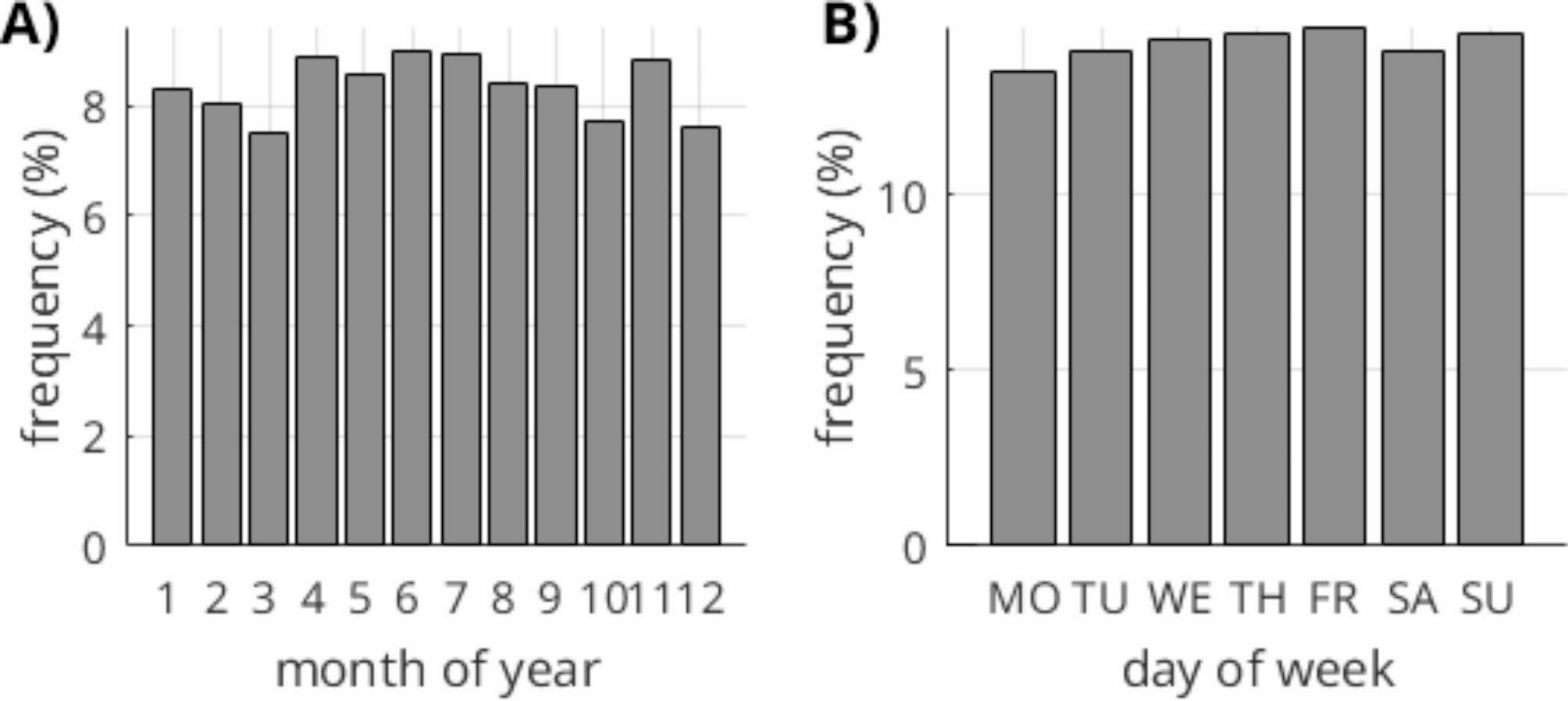
Distribution of scans in a month- (**A**) and weekday-wise (**B**) analysis. There appears to be a slight increase in cases from April to August. The day-of-week distributions are evenly spread throughout the week with no noteworthy reduction on weekends. Frequencies are normalized to the area-under-curve and month-of-year is corrected for different numbers of days per month (as described in the methods section).

The month-of-year and day-of-week distributions are displayed in (Fig. 3). We noted a slight increase in cases from April to August. The day-of-week distributions are relatively uniform throughout the week, with no relevant decrease observed on weekends. However, it should be noted that this data might be influenced by selection bias such as different referral practices during weekends or holidays. A more detailed analysis requires a combination with additional data.

## Discussion

In this proof-of-concept study, we demonstrated that automatically captured DICOM-timestamps can be exploited as an opportunistic means to assess the temporal distribution of imaging of patients with an acute stroke. Among two separate stroke centers, we consistently observed a nonuniform distribution of stroke-related imaging throughout the day.

Various preclinical tests (e.g., RACE, FAST) have been developed to identify patients with vessel occlusions and reduce onset-to-treatment times. Our findings reveal that both the empirical data and fitted parameters indicate a narrower and earlier peak for patients in the LVO/MVO subgroups compared to the NVO group. This may indirectly suggest that patients with vessel occlusions either seek medical attention more quickly and/or are managed within a more streamlined and efficient stroke care workflow. However, the distributions for onset-to-scan and admission-to-scan intervals - more direct measures of treatment timeliness - do not exhibit this difference. However, the latter data were available for only a subset of 347 cases, so further data are needed to explore the underlying reasons in greater detail.

We found that the probabilistic model provided reasonable fits to the data. However, it is important to note that this does not constitute proof of the model’s validity. In this study, the model was primarily employed for illustrative and comparative purposes and the results should not be over-interpreted. It should be emphasized that we had to make several simplifications and assumptions. One limitation is that we neglected a circadian variation of stroke onsets. Circadian variations have been reasoned and examined in several studies^12–18^. It is generally accepted that the stroke frequency is lower during the night and highest in the morning hours. These “early-morning strokes” could be an alternative or additional explanation for the peak around noon observed in our work. In fact, from a mathematical perspective, it is not possible to distinguish between wake-up strokes and early morning strokes, resulting in ambiguities in the model. In our simplified model, we have classified early morning strokes as wake-up strokes, which results in a relatively high proportion of wake-up strokes at around 40%. However, in literature, typically lower values of around 20% are reported^19^.

This ambiguity is also represented in another minor, but noteworthy finding: On visual inspection of the distributions in Fig. 2, one might speculate that there is a small second peak around late afternoon, consistently observable in all the graphs. This finding is in line with a preceding lunchtime nap, a habit that is reported to be common in an estimated 20–40% of the German population^20,21^. The presence of an afternoon peak is also described in the review article on circadian variations^18^. Analog to the morning peak, it might be explained by strokes occurring during the nap, which then manifest as afternoon wake-up strokes, an increased probability of stroke occurrence directly after waking up, or both.

Combined analysis of consistently recorded onset data with DICOM-timestamps might allow for more precise modeling and improved description of the actual circadian variation.

Operational hours might be another confounding factor, but several considerations suggest they play only a minor role: Both participating centers are comprehensive stroke centers with 24/7 staffing, ensuring round-the-clock imaging capacities. Furthermore, imaging times show strong temporal consistency with documented onset times (Fig. 1F), which are not influenced by operational hours. Lastly, if operational hours were a significant confounder, we would expect a noticeably lower proportion of cases managed on weekends than on weekdays. However, our data show no such pattern, suggesting that operational hours do not substantially affect the observed effects.

Despite these limitations in model fitting and details of its interpretation, we have demonstrated that DICOM timestamps can be employed to assess time data in stroke imaging and compare those between different stroke centers. While DICOM timestamps alone do not directly reflect absolute treatment timeliness, comparing them between stroke centers can provide valuable insights into the relative temporal distribution of imaging. DICOM data can be extracted in a retrospective and standardized manner from any PACS around the globe. This could be used as a generalized quality control and optimization measure in the certification procedure of stroke units. Furthermore, by refining the model and combining it with other timestamps, we may be able to gain more scientific insights. To further support this research and encourage collaboration, we have made our full dataset and the programming code for model fitting freely available in a public repository (10.6084/m9.figshare.24501325).

The use of DICOM timestamps for analyzing workflow in healthcare settings has been proposed previously, specifically in the context of acute ischaemic stroke. Goyal et al. performed a detailed analysis of the workflow in acute ischemic stroke and identified a basic set of measurable time intervals, including DICOM timestamps^4^.

While the cited work provides an excellent theoretical analysis, we were able to implement the concept in a practical manner on real-world data from a large, multicentric dataset, including a specific sub-analysis of patients with and without vessel occlusions. The approach provides a unique and valuable perspective on stroke care and highlights the potential of DICOM timestamps.

Our analysis focused on the DICOM tag *StudyTime*. Szczykutowicz et al. examined a broader range of potentially useful DICOM tags on a more granular level to measure the efficiency within an examination^22^. This allows for a more comprehensive assessment of the imaging workflow, such as the intervals between consecutive measurements.

The concept of DICOM tags could even be expanded to measuring the duration from therapy decision based on CT imaging and the start of the actual mechanical thrombectomy intervention as it also provides automatically captured DICOM time-stamps. One might even think of taking advantage of the DICOM infrastructure by acquiring “dummy-images” at key timepoints in the treatment workflow, such as arterial puncture. This could potentially overcome the limitations of error-prone manual recording and the need for additional digital capturing infrastructure.

However, it should be noted that even though the DICOM is in principle highly standardized, there are differences in the interpretation by different vendors, especially tags on the series level. For example, the tag *SeriesTime* might refer to the start of the actual series, or to the timepoint the protocol was selected^7^. Such potential pitfalls must be kept in mind.

Finally, the approach of utilizing automatically captured timestamp data might be extended to other modalities, such as blood draws or other diagnostic procedures, allowing for a more comprehensive analysis of patient management even if there was no dedicated human documentation of the relevant time points.

## Conclusion

In conclusion, we performed an analysis of multicentric real-world data of imaging times in acute ischemic stroke. We demonstrated that the concept is technically feasible. While imaging times alone provide only an indirect measure of treatment timeliness, they represent a crucial piece in the broader assessment of stroke management and might enable standardized, large-scale comparisons between centers.

## Electronic supplementary material

Below is the link to the electronic supplementary material.

Supplementary Material 1

## Data Availability

To further support research, we have made our full dataset and the programming code for model fitting freely available in a public repository (10.6084/m9.figshare.24501325).

## References

[1] Saver, J. L. Time is brain–quantified. *Stroke* **37**, 263–266 (2006). 16339467 10.1161/01.STR.0000196957.55928.ab

[2] Emberson, J. et al. Effect of treatment delay, age, and stroke severity on the effects of intravenous thrombolysis with alteplase for acute ischaemic stroke: a meta-analysis of individual patient data from randomised trials. *Lancet* **384**, 1929–1935 (2014). 25106063 10.1016/S0140-6736(14)60584-5 PMC4441266

[3] Goyal, M. et al. Endovascular thrombectomy after large-vessel ischaemic stroke: a meta-analysis of individual patient data from five randomised trials. *Lancet* **387**, 1723–1731 (2016). 26898852 10.1016/S0140-6736(16)00163-X

[4] Goyal, M. et al. Standardized reporting of workflow metrics in acute ischemic stroke treatment: why and how? *Stroke: Vasc. Intervent. Neurol.* **1**, e000177 (2021). 10.1161/SVIN.121.000177 PMC12512355 41584468

[5] Powers, W. J. et al. Guidelines for the early management of patients with acute ischemic stroke: 2019 update to the 2018 guidelines for the early management of acute ischemic stroke: a guideline for healthcare professionals from the American heart association/American stroke association. *Stroke* **50**, e344–e418 (2019). 31662037 10.1161/STR.0000000000000211

[6] Berenspöhler, S., Minnerup, J., Dugas, M. & Varghese, J. Common data elements for meaningful stroke documentation in routine care and clinical research: Retrospective data analysis. *JMIR Med. Inf.* **9**, e27396 (2021). 10.2196/27396 PMC8548969 34636733

[7] Langer, S. G. DICOM data warehouse: part 2. *J. Digit. Imaging* **29**, 309–313 (2016). 26518194 10.1007/s10278-015-9830-4 PMC4879026

[8] Langer, S. G. A flexible database architecture for mining DICOM objects: the DICOM data warehouse. *J. Digit. Imaging* **25**, 206–212 (2012). 22080292 10.1007/s10278-011-9434-6 PMC3295972

[9] Kellner, E., Rau, A., Demerath, T., Reisert, M. & Urbach, H. Contrast bolus interference in a multimodal CT stroke protocol. *AJNR Am. J. Neuroradiol.*10.3174/ajnr.A7247 (2021). 34413063 10.3174/ajnr.A7247 PMC8562742

[10] Waqas, M. et al. Large vessel occlusion in acute ischemic stroke patients: a dual-center estimate based on a broad definition of occlusion site. *J. Stroke Cerebrovasc. Dis.* **29**, 104504 (2020). 31761735 10.1016/j.jstrokecerebrovasdis.2019.104504

[11] Thompson, H. K., Starmer, C. F., Whalen, R. E. & Mcintosh, H. D. Indicator transit time considered as a gamma variate. *Circ. Res.* **14**, 502–515 (1964). 14169969 10.1161/01.res.14.6.502

[12] Marsh, E. E. et al. Circadian variation in onset of acute ischemic stroke. *Arch. Neurol.* **47**, 1178–1180 (1990). 2241613 10.1001/archneur.1990.00530110032012

[13] Lago, A. et al. Circadian variation in acute ischemic stroke: a hospital-based study. *Stroke* **29**, 1873–1875 (1998). 9731611 10.1161/01.str.29.9.1873

[14] Argentino, C. et al. Circadian variation in the frequency of ischemic stroke. *Stroke* **21**, 387–389 (1990). 2309262 10.1161/01.str.21.3.387

[15] Kelly-Hayes, M. et al. Temporal patterns of stroke onset. *Stroke* **26**, 1343–1347 (1995). 7631334 10.1161/01.str.26.8.1343

[16] Marler, J. R. et al. Morning increase in onset of ischemic stroke. *Stroke* **20**, 473–476 (1989). 2648651 10.1161/01.str.20.4.473

[17] Elliott, W. J. Circadian variation in the timing of stroke onset: a meta-analysis. *Stroke* **29**, 992–996 (1998). 9596248 10.1161/01.str.29.5.992

[18] Fodor, D. M. & Marta, M. M. Perju-Dumbravă, L. Implications of circadian rhythm in stroke occurrence: certainties and possibilities. *Brain Sci.* **11**, 865 (2021). 34209758 10.3390/brainsci11070865 PMC8301898

[19] Peter-Derex, L. & Derex, L. Wake-up stroke: from pathophysiology to management. *Sleep. Med. Rev.* **48**, 101212 (2019). 31600679 10.1016/j.smrv.2019.101212

[20] Ohayon, M. M., Priest, R. G., Zulley, J., Smirne, S. & Paiva, T. Prevalence of narcolepsy symptomatology and diagnosis in the European general population. *Neurology* **58**, 1826–1833 (2002). 12084885 10.1212/wnl.58.12.1826

[21] Meier, U. Das schlafverhalten der deutschen bevölkerung—eine repräsentative studie. *Somnologie* **8**, 87–94 (2004).

[22] Szczykutowicz, T. P. et al. A General framework for monitoring image acquisition workflow in the radiology environment: timeliness for acute stroke CT imaging. *J. Digit. Imaging* **31**, 201–209 (2018). 29404851 10.1007/s10278-018-0055-1 PMC5873477

